# Prevalence and determinant factors of low birth weight in Marrakesh province, Morocco: cross sectorial survey

**DOI:** 10.4314/ahs.v24i2.32

**Published:** 2024-06

**Authors:** Soufiane Elmoussaoui, Kamal Kaoutar, Ahmed Chetoui, Abdeslam El Kardoudi, Fatiha Chigr, Mounir Borrous, Mohamed Najimi

**Affiliations:** 1 Biological Engineering Laboratory, Faculty of Sciences and Techniques, Sultan Moulay Slimane University, Beni Mellal, Morocco; 2 Mohamed VI University Hospital , Marrakesh, Morocco

**Keywords:** Low birth weight, newborn, mothers, Marrakesh, Morocco

## Abstract

**Background:**

Low Birth Weight (LBW) is considered as the marker of infant wellbeing and the fundamental focus of infant health policy. The objective of this survey was to determine the prevalence of LBW and its associated factors in term new borns.

**Methods:**

The data was collected using an interviewer administered questionnaire. Both bivariate and multivariate logistic regression analyses were used to identify factors associated with LBW.

**Results:**

Totally 350 mother–newborn pairs were participated in this study. Out of this, 16.7% of term neonates were found to be LBW. Of note, employed mothers, mothers having birth interval less or equal to two years, women with previous history of low birth weight and mothers living in passive smoking conditions at home during pregnancy were more likely to have low birth weight babies.

**Conclusion:**

The prevalence of LBW in our study could be considered as relatively high. It is recommended that special attention should be given to pregnant mothers to get adequate rest, attentional diet, and antenatal services available and accessible to all pregnant women.

## Introduction

Low Birth Weight (LBW) constitutes a major public health problem. World Health Organization (WHO) defines LBW as the birth weight less than 2500 grams irrespective of gestational age.

Prevalence of Low birth weight across the globe accounts for 15.5%, which means that each year from 130 million annual births, 20 million is low birth weight^1^. Although there is variation in the number of low birth weight babies across regions, low and middle-income countries took a high figure particularly in most vulnerable populations^2^. In 1995, 53% of deaths that are occurring in children under 5 years of age were associated with LBW^3^. Birth weight has emerged as the pointer of infant well-being and the fundamental focus of infant health policy^4^. LBW predisposes newborns to many health disarrays like underweight, stunting, hypoglycemia, hypothermia, mental retardation, physical, and neurodevelopment problems which results in high rates of morbidity and mortality^5^,^6^. Being low birth weight is associated with impairment of growth and development and also chronic disease later in life^7^. LBW is typically due to two causes sometimes associated: prematurity (birth occurring before the 37^th^ week of amenorrhea) and intrauterine growth retardation IUGR (weight and or size too small for gestational age by compared to the reference values). For this, it is considered as a multifactorial health problem affecting a priority population. Furthermore, LBW constitutes a valuable public health indicator of maternal health, nutrition, health care delivery, and poverty as LBW babies are at a higher risk of illness and death and shortly after birth and non-communicable disease in the life course^8^. Its incidence is a perinatal health indicator as LBW infants are 20 times more likely to develop complications and die in comparison to normal weight babies^9^. On the other hand, LBW babies are in the potential risk of cognitive deficits, motor delays, cerebral palsy, and other behavior and psychological problem^10^-^14^. The household cost, as well as health system costs could be saved by reducing the burden of LBW 15. IUGR is the outcome of insufficient uterine–placental perfusion and fetal nutrition affecting the overall anthropometric parameter of the fetus.

Prevention is possible through targeted interventions on proven modifiable factors efficiency in several countries around the world. Prevalence of LBW across the globe accounts for 15.5%, which means that each year from 130 million annual births, 20 million is low birth weight^16^. In some developed countries, the proportion of LBW ranges from 2-3% whereas in many developing countries the proportion of LBW range 25-30%^17^,^18^. Morocco is placed 126th in the report on development of UNICEF with a very high LBW rate (12%) and neonatal mortality (36 %o) despite national programs developed for maternal and child health. This prevalence of LBW in Morocco is higher than those recorded in Middle East North African countries as Algeria (7%), Tunisia (7%) and Jordan (10%). LBW tells fetal exposure to risk factors such as maternal unfavorable socioeconomic conditions, smoking habit, malnutrition, and diseases as well as lack of attention to prenatal care and delivery 19-21. It also has a crucial role in estimating whether the newborn is at risk of death and disease during their neonatal period^22^. Concerning the situation in Morocco, a part of a general UNICEF report (data collected from Moroccan health ministery), there is no survey or investigation of thisspect in Morocco. Therefore, determining the magnitude and identifying the risk factors for LBW have the potential role in formulating strategies for reducing LBW in Morocco. In this context, the objectives of this study were to assess LBW and its associated factors among mothers in Marrakesh province.

## Methods

### Study area

This study was conducted in Marrakesh Morocco. According to data from the Moroccan General Census of Population and Housing of 2014, Marrakesh includes a population of nearly 928850 habitants^23^,^24^.

### Study design and sample

This was a cross-sectional study conducted in Marrakesh, Morocco.

### Sample size

This was calculated using single proportion formula by considering the following assumption the prevalence of LBW among term new borns was unknown and taken as 50% , margin of error of 5%, confidence level of 95%, 10% non-response rate, and a design effect of 1,5. The final sample size was 320 25. Subjects were mothers with newborns living in Marrakesh. Mothers who had multiple births were excluded, only 6 women were excluded. All in all, 305 wre completed, with a valid response rate of 96%. Nine mothers refused to participate because they claimed that the interview will take too much time. To select study participants, a multistage sampling technique was used. Data gathering was conducted in 2020.

### Data collection

A pretested, structured questionnaire was used to collect data. Five data collectors (clinical nurses) and two supervisors (pediatrician) were recruited for the task. To maintain consistency, the questionnaire was first translated from French to Arabic, the native language of the study area. It contains socio-demographic, environmental characteristics and healthcare conditions.

Birth weight of naked newborn was measured within an hour of delivery before significant postnatal weight loss has occurred using a balanced infant scale. A professor of pediatrics and two pediatricians assessed the validity of the questionnaire. Pre-testing was completed on 5% of the total respondents to determine whether the questionnaire was understandable, and corrections were made progressively. The face-to-face interviews required almost 20 min.

### Statistical analysis

A descriptive analysis was performed using means and standard deviations (SD). To estimate the significance of the differences observed between the means, the Chi2 test was used for categorical variables. Bivariate logistic regression analysis was done to decide whether there is an association between low birth weight and different factors to select nominee variables for multivariate logistic regression. The odds ratio with 95% confidence intervals (CI) was calculated to distinguish the occurrence and strength of associations, and statistical significance was affirmed if p <0.05. The statistical treatment of data was performed using SPSS software PC-FR, version 19.

### Ethical consideration

The study was performed in accordance to declaration of Helsinki. Due to lockdown, universities were closed; hence study protocol was approved from Hospital board (SAA N°252/2020). Study questionnaire contained consent portion that stated purpose, nature of survey, study objectives, volunteer participation, declaration of confidentiality and anonymity.

## Results

### Sociodemographic characteristics, Obstetric, nutrition-related characteristics of mothers and sex of neonates

A total of 305 mothers participated in this study which gives a response rate of 95.3%. We notice the presence of a percentage of 68.9% of mothers aged less than 30 years and that the studied sample was equally devised between urban (49.8%) and rural areas (50.2%). The illiteracy rate among the women surveyed was 16.7% whereas 32.8% of mothers had a primary school education level, 44.6% a secondary school and 5.9% a higher education. Employment was very low since 95.7% of the women interviewed were housewives. Among the mothers studied, the majority of them were married (94.1%), and the rest were unmarried (3.9%) or divorced (2%). The predominant ethnic group was Arabic with 63.6% followed by Amazigh (36.4%). Concerning monthly family income among the study population, 49.3% of families have a lower income, 47.6% have a medium income and only 3.1% have a higher income (Table 1).

**Table 1:** Socio-demographic characteristics, obstetric, nutrition-related characteristics of mothers and sex of neonates

	Category (n=320)	Frequency	Percent (%)
**Mother's age groups (in years)**	< 20	27	8.9
	20-30	183	60.0
	31 and above	95	31.1
**Area of residence**	Urban	152	49.8
	Rural	153	50.2
**Literacy**	No formal education	51	16.7
	Primary school	100	32.8
	Secondary school	136	44.6
	Higher education	18	5.9
**Mother's professional activity**	Housewife	292	95.7
	Paid worker	13	4.3
**Marital status**	Married	287	94.1
	Divorced	06	2.0
	Unmarried	12	3.9
**Household income**	Lower	146	49.3
	Medium	141	47.6
	Higher	09	3.1
**Ethnicity**	Arab	194	63.6
	Amazigh	111	36.4
**Sex of neonate**	Male	154	50.5
	Female	151	49.5
**Birth interval**	< 2 yrs	22	11.7
	2-3yrs	73	38.8
	4 and above	93	49.5
**Parity**	Primiparous	114	38.4
	Multiparous	188	61.6
**Mode of delivery**	Vaginal/normal	202	66.2
	Caeserian section	103	33.8
**ANC follow-up**	No follow-up	03	1.0
	1-3 follow-up	167	54.7
	≥4 follow-up	135	44.3
**Dietary counseling during pregnancy**	Yes	109	35.7
	No	196	64.3
**Foliate supplementation**	Yes	24	7.9
	No	281	92.1
**Iron supplementation**	Yes	238	78.0
	No	67	22.0
**Previous history of LBW**	Yes	57	28.2
	No	145	71.2
**Calcium supplementation**	Yes	06	2.0
	No	299	98.0
**History of contraceptive use**	Yes	193	63.3
	No	112	36.7
**GD**	Yes	36	11.8
	No	269	88.2
**History of abortion**	Yes	95	31.1
	No	210	68.9
**Anemia**	Yes	124	40.7
	No	181	59.3
**GH**	Yes	21	6.9
	No	284	93.1
**Infection**	Yes	36	11.8
	No	269	88.2
**Walk for 30 munites**	Yes	242	79.3
	No	63	20.7
**Smoking habit**	Yes	03	1.0
	No	302	99.0
**Smoking by family member**	Yes	85	27.9
	No	220	72.1
**Support from husband in day to day activities**	Yes	160	55.2
	No	130	44.8
**Type of food use**	As usual	139	45.6
	Addition food (any group)	84	27.5
	Lower food (any group)	82	26.9

*GD: Gestational diabetes; GH: Gestational hypertension*

Regards infant sex, 49.5% were females and almost one half (40.5%) of children were first in birth order. 35.7% of mothers were counseled concerning dietary and the primiparous women accounted for 38.4% of the sample, while multiparous mothers accounted for 61.6%. Only 11.7% of mothers gave birth to the current newborn less than two years (< 24 months) after previous childbirth. When delivery is considered, a majority of women delivered vaginally (66.2%) (Table 1). Of interest, only one half (55.2%) of these pregnant women questioned have been supported by their husband. Concerning smoking status of participants (99%) did not smoke any form of cigarette but with regard to passive smoking, third (33.2%) of mothers have a family member who had the habit of smoking cigarettes during pregnancy inside the house. The majority of participants had their meal three times a day (73.1%) and 27.5% had included additional food groups in their meal at the time of pregnancy. When physical activity is considered, 79.3% of participants walked daily for 30 minutes during pregnancy. Concerning antenatal Care (ANC) visit as per the protocol of the government of Morocco, the majority (78%) of participants had taken iron tablets during pregnancy, 7.9% had taken foliate supplementation and only 2% of participants had taken calcium supplementation. Among the sample surveyed, 57 mothers (28.2%) had a low birth weight in the previous pregnancy. For health problems, Ninety five (31.1%) mothers had a history of abortion, 21 (6.9%) hypertension, 36 (11.8%) diabetes, 36 (11.8%) infection and 124 (40.7%) had anemia during the current pregnancy.

### Prevalence and associated factors with low birth weight

In this study, 16.7% of term neonates were found to be LBW. In multi variable logistic regression; maternal occupation, birth interval, previous history of LBW and smoking by family member were statistically associated with low birth weight at P-value < 0.05.

Employed mothers were 17 times more likely to have low birth weight babies as compared to mothers who had no employment AOR=17.409(4.580-35.139). Mother who gave birth with birth interval less or equal to two years were twelve times more likely to have low birth weighed babies as compared to those who have three years and above AOR=12.50(2.902-5.330). Furthermore, women who had previous history of low birth weight had 15 times higher odds ratio of delivered low birth weight baby than their counterparts AOR =15.880(4.5-56.0). Finally, smoking by a family member during pregnancy were about 3 times more likely to have low birth weighed babies AOR: 3.259(1.918-11.589) (Table 2).

**Table 2:** Factors associated with LBW

Variables	LBW		x^2^ test	Multivariate analysis	
	Yes n (%)	No n (%)	p-value	Adjusted OR (95% CI)	p-value
**Mother's age groups (in years)**				…………………………………	…………………
< 20	3(11.1)	24(88.9)	0.235		
20-29	36(19.7)	147(80.3)			
30 and above	12(12.6)	83(87.4)			
**Area of residence**				…………………………………	…………………
Urban	27(17.8)	125(82.2)	0.627		
Rural	24(15.7)	129(84.3)			
**Mother's professional activity**					
Paid worker	09(69.2)	04(30.8)	**<0.001**	17.409(4.580-35.139)	**0.005**
Housewife	42(14.4)	250(85.6)		1	
**Ethnicity**				…………………………………	…………………
Arabe	33(17.0)	161(83.0)	0.858		
Berbère	18(16.2)	93(83.8)			
**Parity**				…………………………………	…………………
Primiparous	15(12.8)	102(87.2)	0.150		
Multiparous	36(19.1)	152(80.9)			
**Birth interval**					
< 2 yrs	06(27.3)	16(72.7)	**<0.001**	12.50(2.902-5.330)	**0.005**
2-3yrs	03(4.1)	70(95.9)		1.048(0.156-7.045)	0.961
3 and above	27(29.0)	66(71.0)		1	
**Mode of delivery**				…………………………………	…………………
Vaginal/normal	36(17.8)	166(82.2)	0.471		
Caeserian section	15(14.6)	88(85.4)			
**ANC follow-up**				…………………………………	…………………
No follow-up	0(0.0)	03(100.0)	0.688		
1-3 follow-up	27(16.2)	140(83.8)			
≥4 follow-up	24(17.8)	111(82.2)			
**Dietary counseling during pregnancy**					
Yes	12(11.0)	97(89.0)	**0.046**	0.294(0.059-1.472)	0.136
No	39(19.9)	157(80.1)		1	
**Iron supplementation**				…………………………………	…………………
Yes	39(16.4)	199(83.6)	0.768		
No	12(17.9)	55(82.1)			
**Previous history of LBW**					
Yes	21(36.8)	36(63.2)	**<0.001**	15.880(4.5-56.0)	**<0.001**
No	15(10.3)	130(89.7)		1	
**Calcium supplementation**				…………………………………	…………………
Yes	0(0.0)	6(100.0)	0.268		
No	51(17.1)	248(82.9)			
**History of contraceptive use**				…………………………………	…………………
Yes	30(15.5)	163(84.5)	0.470		
No	21(18.8)	91(81.3)			
**Anemia**				…………………………………	…………………
Yes	18(14.5)	106(85.5)	0.393		
No	33(18.2)	148(81.8)			
**GH**				…………………………………	…………………
Yes	6(28.6)	15(71.4)	0.132		
No	45(15.8)	239(84.2)			
**History of abortion**				…………………………………	…………………
Yes	18(18.9)	77(81.1)	0.483		
No	33(15.7)	177(84.3)			
**Marche 30 munites**				…………………………………	…………………
Yes	39(16.1)	203(83.9)	0.579		
No	12(19.0)	51(81.0)			
**Smoking habit**				…………………………………	…………………
Yes	0(0.0)	3(100.0)	0.435		
No	51(16.9)	251(83.1)			
**Smoking by family member**					
Yes	24(28.2)	61(71.8)	**0.001**	3.259(1.918-11.589)	**0.03**
No	27(12.3)	193(87.7)		1	
**Support from husband in day to day activities**					
Yes	18(11.3)	142(88.8)	**0.026**	0.604(0.183-1.992)	0.408
No	27(20.8)	103(79.2)		1	
**Food frequency per day**				…………………………………	…………………
Twice	12(25.0)	36(75.0)	**0.140**		
Thrice	36(16.1)	187(83.9)			
More	03(8.8)	31(91.2)			
**Type of food use**					
As usual	18(12.9)	121(87.1)	**0.002**	1.684(0.292-9.702)	0.559
Addition food (any group)	09(10.7)	75(89.3)		1.684(0.026-1.861)	0.165
Lower food (any group)	24(29.3)	58(70.7)			

## Discussion

To our knowledge, this investigation constitutes a first detailed one concerning LBW in Morocco. This cross-sectional study is aimed to assess the prevalence of LBW and its associated factors of Moroccan newborn in Marrakesh in center of Morocco.

World Health Organization (WHO) defines low birth weight as a birth weight of an infant 2499 gram or less irrespective of gestational age 26. In our study, the prevalence of LBW was 16.7%. It was relatively high than national prevalence reported by UNICEF (15%)^27^. This difference might be due to the previous studies were carried out in specialized hospitals where many of the pregnant women were referred from peripheral hospitals because of high risk pregnancy.

Concerning this context, our finding was consistent also with previous studies done in Ethiopia (17.3%) 28. however, this finding was higher than those reported in North Africa: Algeria (7%), Tunisia (7%) 29 and sub Saharian African countries like Gambia 10.5% 30, Nigeria (14.1%) 31, Zimbabwe 12.9% 32, Kenya (12.3%) 33, and Europe like Spain (6.9%), United Kingdom (8%), France (7%) 34, America like United States (8%) and Canada (6%) 35,36 and Asia like Jordan(10%) 28 Iran 8,8% 37 and Malaysia (12.6%) 38. On the other hand, our finding was lower than that found in Tanzania (22.30%) 39 and in Debre Markos referral hospital Ethiopia (26.3%)^40^. Pakistan19% 41, and India (22.9%)^42^.

For the case, the weight of newborns varies from 600g to 2480g with an average of 1706.86g (SD = 499.42g), while for the controls, the weight of newborns varies from 2500g to 5100gwith an average of 3383.01g (SD = 470.38g).

This calculated average value is lower than those found in Morocco by Amor (1989) 43, Baali (1997)^44^, Belkeziz (2000)^45^ and Elkhoudri (2014) 46 for births in the city of Marrakech and which are respectively 3300g; 3350g; 3300g and 3277g. Nevertheless, there is a slight difference between the average of LBW of those studies andurs.

The possible reason for this difference might be a variation in study time or mother's nutritional status and also the health professional's commitment to antenatal care service provision especially on dietary counseling during pregnancy.

In our study, maternal occupation, Birth interval, previous history of LBW and smoking by a family member were statistically associated with low birth weight at P-value < 0.05.

Indeed, mother occupation was significantly associated with low birth weight of newborns. Employed mothers were 17 times more likely to have low birth weighted babies as compared to mothers who didn't employed AOR=17.409(4.580–35.139). This might lead employed mothers to be psychologically stressed on work due to the hardness of their work conjugated to their responsibilities in house. This affects greatly their health care seeking behavior.

Mothers who gave birth with birth interval less or equal to two years were 12 times more likely to have low birth weighed babies as compared to those who have three years and above AOR=12.50(2.902-5.330). This might be due to shorter birth. This later might increase the life risk for mothers related to pregnancy and delivery complications. It may directly or indirectly affects mothers' health, economic and social status during pregnancy.

Previous history of low birth weight explained a significant association with low birth weight. Indeed, women having previous history of LBW had higher odds to have delivery of LBW neonates than women who did not have previous history AOR=15.880(4.5-56.0). This finding was similar with those found in studies conducted in Nigeria 47 and Japan 48.

In our study, Smoking by family member during pregnancy were about 3 times more likely to have low birth weighed babies AOR: 3.259(1.918-11.589). This finding is consistent with the findings of studies done in Bangladesh and Turkey 50-51.

Smoking during pregnancy had a negative effect on the growth and development of the fetus because of chemical substances present in it. Nicotine present in the cigarette cause vasoconstriction resulting in the low oxygen flow to the fetus and Carbon monoxide forms carboxy hemoglobin, which inhibits the oxygen release to fetal tissues 49. Smoking is considered also as a factor of sudden infant death syndrome (SIDS) 52. In Morocco, SIDS cases are not diagnosed as that and in final; this could explain at least partially this high rate.

## Conclusion

The prevalence of LBW in our study was relatively high than national prevalence reported by UNICEF.

This study showed that Socio-economic (maternal occupation), cultural characteristics (Birth interval), and health status (previous history, smoking) were risk factors for low birth weight in the study areas. This study found that maternal occupation, Birth interval, previous history of LBW and smoking by family member were statistically associated with LBW at P-value < 0.05. Prevention of low birth weight is an important intervention to reduce neonatal death. To reduce LBW neonate health care providers need to work to early detect and manage risk factors that cause LBW. These findings contribute to the growing literature on the influence of maternal and paternal socio-economic, cultural factors and health status on LBW in resource-constrained settings.

It is advisable that those health care providers need to work in the community on the importance of negative effects of passive smoking during pregnancy and the consequence of birth interval less than 2 years, history of LBW outcomes to the current pregnancy through focused antenatal care on the prevention of LBW newborns. The problem of LBW in Morocco needs focused attention, and research requires innovative strategies to attempt to identify protective factors among women who are at high risk.

## References

[1] WHO/UNICEF (2009). Prematurity and Low Birth Weight.

[2] United Nations Children's Fund and World Health Organization (2004). Low Birth Weight: Country, Regional And Global Estimates.

[3] United Nations Administrative Committee on Coordination (2000). Fourth report on the world nutrition situation.

[4] Badshah S, Mason L, McKelvie K, Payne R, Lisboa PJ (2008). “Risk factors for low birth weight in the public-hospitals at Peshawar, NWFP-Pakistan”. BMC Public Health.

[5] Shrimpton R (2003). “Preventing low birth weight and reduction of child mortality”. Transactions of the Royal Society of Tropical Medicine and Hygiene.

[6] Hack M, Klein NK, Taylor HG (1995). “Long-term develop-mental outcomes of low birth weight infants”. The Future of Children.

[7] Chiarotti A, Puopolo M, Gissler M, Sihvonen E, Hemminki K (2001). “Efects of socio-environmental factors on neurocognitive performance in premature or low birth weight preschoolers”. Annali dell'Istituto Superiore di Sanit'a.

[8] Agbozo F, Abubakari A, Der J, Jahn A (2016). Prevalence of low birth weight, macrosomia and still birth and their relationship to associated maternal risk factors in Hohoe Municipality, Ghana. Midwifery.

[9] UNICEF, WHO (2004). Low Birth Weight: Country, regional and global estimates.

[10] Mathewson KJ, Chow CH, Dobson KG, Pope EI, Schmidt LA, Van Lieshout RJ (2017). Mental health of extremely low birth weight survivors: A systematic review and meta-analysis. Psychological Bulletin.

[11] Al Hazzani F, Al-Alaiyan S, Hassanein J, Khadawardi E (2011). Short-term outcome of very low-birth-weight infants in a tertiary care hospital in Saudi Arabia. Annals of Saudi Medicine.

[12] Chang H-Y, Sung YH, Wang SM, Lung HL, Chang JH, Hsu CH (2015). Short and Long-Term Outcomes in Very Low Birth Weight Infants with Admission Hypothermia. PloS One.

[13] Fitzgibbons SC, Ching Y, Yu D, Carpenter J, Kenny M, Weldon C Mortality of necrotizing enterocolitis expressed by birth weight categories. Journal of Pediatric Surgery.

[14] Fan RG, Portuguez MW, Nunes ML (2013). Cognition, behavior and social competence of preterm low birth weight children at school age. Clinics.

[15] Sicuri E, Bardají A, Sigauque B, Maixenchs M, Nhacolo A, Nhalungo D (2011). Costs Associated with Low Birth Weight in a Rural Area of Southern Mozambique. PloS one.

[16] WHO/UNICEF (2009). Prematurity and Low Birth Weight.

[17] Blanc AK, Wardlaw T (2005). “Monitoring low birth weight: an evaluation of international estimates and an updated estimation procedure”. Bulletin of the World Health Organization.

[18] WHO AJ (1976). Technical Report Series.

[19] Moser K, Li L, Power C (2003). “Social inequalities in low birth weight in England and Wales: trends and implications for future population health”. Journal of Epidemiology & Community Health.

[20] Salihu HM, Shumpert MN, Aliyu MH, Kirby RS, Alexander GR (2005). “Smoking-associated fetal morbidity among older gravidas: a population study”. Acta Obstetricia et Gynecologica Scandinavica.

[21] Torres-Arreola LP, Constantino-Casas P, Flores-Hernandez S, Villa-Barragan JP, Rendon-Macias E (2005). “Socioeconomic factors and low birth weight in Mexico”. BMC Public Health.

[22] Vettore M V, da Gama S G N, Lamarca G D A, Schilithz A O C, Leal MDC (2010). “Housing conditions as a social determinant of low birth weight and preterm low birth weight”. Revista de Saude Publica.

[23] High Commission for Planning (HCP) of Morocco (2014). General census of population and housing in the region of Casablanca-Settat.

[24] Habibi M, Laamiri FZ, Aguenaou H, Doukkali L, Mrabet M, Barkat A (2017). Evaluation of the infant and young child feeding index among Moroccan children. J Med Surg Res.

[25] Kish L (1965). Survey Sampling.

[26] Lawn JE, Gravett MG, Nunes TM, Rubens CE, Stanton C, The GAPPS Review Group (2010). “Global report on preterm birth and still birth (1 of 7): defnitions, description of the burden and opportunities to improve data”. BMC Pregnancy and Childbirth.

[27] United Nation Children's Fund (2008). The state of the world's children 2009: Maternal and Newborn Health.

[28] Endalamaw A, Engeda EH, Ekubagewargies DT, Belay GM, Tefera MA (2018). “Low birth weight and its associated factors in Ethiopia: a systematic review and meta-analysis”. Italian Journal of Pediatrics.

[29] Wardlaw Tessa M (2004). Low Birth weight: Country, regional and global estimates.

[30] Abdou J, Johanne S, Siri V (2011). Maternal and obstetric risk factors for low birth weight and preterm birth in rural Gambia: a hospital-based study of 1579 deliveries. Open J Obstet Gynaecol.

[31] Awoleke JO (2012). Maternal risk factors for low birth weight babies in Lagos, Nigeria. Arch Gynecol Obstet.

[32] Ticconia C, Arpino C, Longo B (2005). Prevalence and risk factors for low birthweight in Northern Zimbabwe. Int J Gynaecol Obstet.

[33] Muchemi OM, Echoka E, Makokha A (2015). Factors associated with low birth weight among neonates born at Olkalou district hospital, central region, Kenya. Pan Afr Med J.

[34] WHO/UNICEF (2004). Low birth weight. Country regional and global estimates.

[35] Martin JA, Hamilton BE, Osterman MJ, Driscoll AK, Matthew TJ (2017). “Births: final data for 2015”. National Vital Statistics Reports.

[36] Statistics in Canada (2018). “Low birth weight newborn in Canada: health fact sheet, 2000 to 2013”.

[37] Golestan M, Akhavan KS, Fallah R (2011). Prevalence and risk factors for low birth weight in Yazd, Iran. Singapore Med J.

[38] Boo NY, Lim SM, Koh KT, Lau KF, Ravindran J (2008). Risk factors associated with low birth weight infants in the Malaysian Population. Med J Malaysia.

[39] World Health Organization (1980). “Division of Family Health. The incidence of low birth weight: a critical review of available information”. World Health Statistics Quarter Reports.

[40] Dilie A, Chanie H (2018). “Prevalence of low birth weight and associated factors among women delivered in debre markos referral hospital, East Gojam, Ethiopia, 2017”. Journal of Health, Medicine and Nursing.

[41] Najmi RS (2000). Distribution of birth weights of hospital born Pakistani infants. J Pak Med Assoc.

[42] Chandra SM, Vijaya AN, Maheshwar DM (2012). Factors Affecting Birth Weight of a Newborn - A Community Based Study in Rural Karnataka, India. PLoS One.

[43] Amor H (1989). Weight growth and evaluation of the nutritional status of infants from birth to 2 years in the prov-ince of Marrakech (Morocco). 3rd cycle thesis.

[44] Baali Amor H, Pagezy H (1997). Weight growth from birth to two years of children in the province of Marrakech. IREMAM Notebooks.

[45] Belkeziz N Croissance pondérale et état nutritionnel des enfants Marocains de la naissance à l'âge de deux ans 2000. State thesis.

[46] Elkhoudri N, Baali A (2015). Prevalence and Determinants of Low Birth Weight: A Case-Control Study in Marrakesh (Morocco). Iran J Public Health.

[47] Hirut M, Kebebush Z (2016). “Magnitude and factors associated with low birth weight among new born in selected public hospitals of addis ababa, ethiopia”. Global Journal of Medical Research.

[48] Viengsakhone L, Yoshida Y, Harun-Or-Rashid M, Sakamoto J (2010). “Factors affecting low birth weight at four central hospitals in Vientiane, Lao PDR”. Nagoya Journal of Medical Science.

[49] Lambers DS, Clark KE (1996). The maternal and fetal physiologic effects of nicotine. Seminars in Perinatology.

[50] Hosain GM, Chatterjee N, Begum A, Saha SC (2006). Factors associated with low birth weight in rural Bangladesh. Journal of Tropical Pediatrics.

[51] Atessahin E, Pirincci E (2015). Risk Factors Associated with Low Birth Weight Infants Bornin Elazig, Eastern of Turkey. Iranian Journal of Public Health.

[52] Mitchell EA, Ford RP, Stewart AW, Taylor BJ, Becroft DM, Thompson JM, Scragg R, Hassall IB, Barry DM, Allen EM (1993). Smoking and the sudden infant death syndrome. Pediatrics.

